# Adding Insult to Injury: When Atrial Fibrillation Encounters Left Bundle Branch Block

**DOI:** 10.31083/j.rcm2512429

**Published:** 2024-12-03

**Authors:** Dong-sheng Zhao, Nishant Yadav, Yan Dong, Qiu-shi Chen, Di Yang, Feng-xiang Zhang

**Affiliations:** ^1^Department of Cardiology, The First Affiliated Hospital with Nanjing Medical University, 210029 Nanjing, Jiangsu, China; ^2^Department of Cardiology, The Second Affiliated Hospital of Nantong University, 226019 Nantong, Jiangsu, China

**Keywords:** atrial fibrillation, left bundle branch block, arterial embolism events, mortality, MIMIC-III database

## Abstract

**Background:**

It is not uncommon that atrial fibrillation (AF) coexists with left bundle branch block (LBBB). Whether LBBB is an independent predictor of poor prognosis in AF patients remains undetermined. This study aims to investigate the impact of LBBB on the AF-related outcomes in non-valvular AF patients.

**Methods:**

The clinical data of AF patients were collected from the Medical Information Mart for Intensive Care-III (MIMIC-III) database. The frequencies of acute arterial embolism events (AEE) and in-hospital cardiac death were compared between the non-LBBB and LBBB groups. And, their 1-year mortality was assessed through a survival analysis model. Additionally, the two groups were matched in a 1:2 ratio by a propensity score matching (PSM) method according to the CHA_2_DS_2_VASc score and AF type.

**Results:**

5051 patients diagnosed with non-valvular AF without apparent structural heart disease were enrolled in this study, among them, there were 65 with LBBB which had more AEE (13.8% vs 6.8%, *p* = 0.04). After PSM, with balanced CHA_2_DS_2_VASc score and AF type, LBBB was still related with AEE (13.8% vs 3.8%, *p* = 0.02) significantly, and it was also independent of heart failure (HF) (odds ratios (OR) 6.38, 95% confidence intervals (CI) [1.10, 36.93], *p* = 0.04). LBBB was also correlated with in-hospital cardiac death (OR 5.33, 95% CI [1.01, 28.28], *p* = 0.04). And, the LBBB patients had a lower 1-year survival rate in the subgroup of HF (67.6% vs 83.0%, *p* = 0.06).

**Conclusions:**

The LBBB was an independent risk factor of AEE and related to in-hospital cardiac death and 1-year all-cause mortality in this non-valvular AF cohort from MIMIC-III.

## 1. Introduction

Atrial fibrillation (AF) is the most frequent arrhythmia being linked to an increased risk of arterial embolism events (AEE), heart failure (HF) and mortality [1]. At present, the CHA_2_DS_2_VASc score is the most commonly used stroke risk assessment tool in non-valvular AF patients with AF [2], and it has also been found to be a powerful predictor of all-cause death [3, 4, 5]. However, there remains a challenge and pitfalls that its applicability to certain populations is limited, in addition, the discriminatory ability in any given individual is moderate at best [6]. Though left bundle branch block (LBBB) is uncommon in the general population (<1% prevalence), its incidence increases with age, reported in up to 5% of octogenarians [7]. Additionally, LBBB and AF may coexist in some patients, particularly those with advanced cardiac disease [8]. LBBB leading to intraventricular and interventricular mechanical dyssynchrony is the etiology, deterioration, and mortality factor of HF [9]. Until now, few studies on the additive effect of LBBB on worsening prognosis in AF patients have been published, and the results are conflicting [10, 11]. In the present study, we intend to investigate whether LBBB itself exacerbates the outcome of AF including AEE, in-hospital death and 1-year all-cause mortality by retrieving the data from Medical Information Mart for Intensive Care-III (MIMIC-III).

## 2. Materials and Methods

### 2.1 Study Design and Data Resource

This is a single-center retrospective cohort study with all the relevant data collected from MIMIC-III (version 1.4, Boston, MA, USA). MIMIC-III is an open and freely accessible database including the in-hospital and follow-up information of over 50,000 critically ill patients at the Beth Israel Deaconess Medical Center (BIDMC) in Boston from 2001 to 2012 [12] such as demographics, vital sign measurements, laboratory tests, procedures, medications, caregiver notes, imaging reports and mortality records. The discharge diagnoses were coded according to the International Classification of Diseases, Ninth Revision (ICD-9). We conducted the cross-sectional study comparing the incidence of AEE, HF, in-hospital mortality and 1-year all-cause mortality between the LBBB and non-LBBB groups in idiopathic AF patients from MIMIC-III. To balance the effect of potential confounders, we performed 1:2 propensity score matching (PSM) according to the CHA_2_DS_2_VASc score and AF type between the two groups. The establishment of the MIMIC-III database was approved by the Institutional Review Boards of the Massachusetts Institute of Technology (Cambridge, MA, USA) and BIDMC, and consent was obtained for the original data collection. Therefore, the ethics approval statement and the requirement for informed consent were waived. The study was conducted in accordance with the principles of the Declaration of Helsinki (as revised in 2013).

### 2.2 Study Population

All the patients with the diagnosis of AF using ICD-9 codes at the first admission on the MIMIC-III database were included. The exclusion criteria were as follows: (1) age less than 18 years old; (2) valvular heart disease; (3) cardiomyopathy: hypertrophic cardiomyopathy (HCM), dilated cardiomyopathy (DCM), ischemic cardiomyopathy (ICM), restrictive cardiomyopathy (RCM), alcoholic cardiomyopathy and other unspecified cardiomyopathy; (4) patients with artificial pacemaker ventricular rhythm; (5) suspected new onset or intermittent LBBB; (6) patients with malignant tumor(s).

### 2.3 Data Extraction

Structured query language with PostgreSQL (version 9.4.6, https://www.postgresql.org/) was used to extract following data: (1) baseline demographic variables including age, sex, body mass index (BMI), the status of nicotine dependence and alcohol abuse; (2) the medications on admission (antiarrhythmic agents, oral anticoagulants, angiotensin converting enzyme inhibitors/angiotensin receptor blocker [ACEI/ARB] and beta-blocker); (3) the treatment regimen of AF including rhythm or rate control and the in-hospital electrocardiography; (4) AF-related outcome: AEE, HF and mortality; (5) the comorbidities: hypertension, diabetes mellitus (DM), coronary artery disease (CAD), stroke, transient ischemic attack (TIA), chronic kidney disease; (6) the initial laboratory tests including blood glucose, hemoglobin, troponin T, creatinine, urea nitrogen; (7) the left ventricular ejection fraction (LVEF) acquired by transthoracic or transesophageal echocardiography. The CHA_2_DS_2_VASc score was calculated from clinical data. AF type was defined as non-persistent AF including paroxysmal and rhythm-controlled AF and persistent AF on rate control medication. 1:2 matching (LBBB group vs non-LBBB group) according to CHA_2_DS_2_VASc score and AF type without replacement was performed using a nearest neighbor matching algorithm, with a fixed caliper width of 0.05 [13].

### 2.4 Statistical Analysis

Categorical variables are presented as total number and percentage, and the intergroup difference was analyzed using Pearson’s chi-square test or Fisher’s exact test. The distribution of continuous variables was examined using the Kolmogorov-Smirnov test. The normally distributed data was presented as mean (standardized differences [SD]) and tested by the Student *t* test. The non-normal distribution data was presented as median (quartiles) and tested by the Mann–Whitney U test. The multivariate logistic regression analysis was used to determine independent risk factors of AEE and in-hospital cardiac death presented as odds ratios (OR) and 95% confidence intervals (CIs). For AEE, because there was no variable except LBBB which had statistical significance (*p* < 0.05) in the univariate analysis, we included all the variables with a significant difference between the two groups (the non-LBBB group and LBBB group) in the multivariate analysis. For in-hospital cardiac death, all the variables with significant associations emerging from univariate analysis were included. The estimated 1-year survival rates were calculated using the Kaplan-Meier analysis, and then compared between the two groups using the log-rank statistics. The mortality factors investigation was conducted using the univariate and multivariate Cox proportional regression models presented as hazard ratios (HR) and 95% CIs. The variables with *p* < 0.10 in the univariable Cox regression analysis entered in the multivariable model. The clinically relevant parameters were not included simultaneously.

A two-tailed *p* < 0.05 was considered statistically significant. The statistics toolbox included R software (version 3.6.1; The R Project for Statistical Computing, Auckland, New Zealand; http://www.r-project.org) and SPSS software (version 22.0; IBM Corporation, St. Louis, MO, USA).

## 3. Results

### 3.1 Characteristics of Patients

**Fig. 1.**
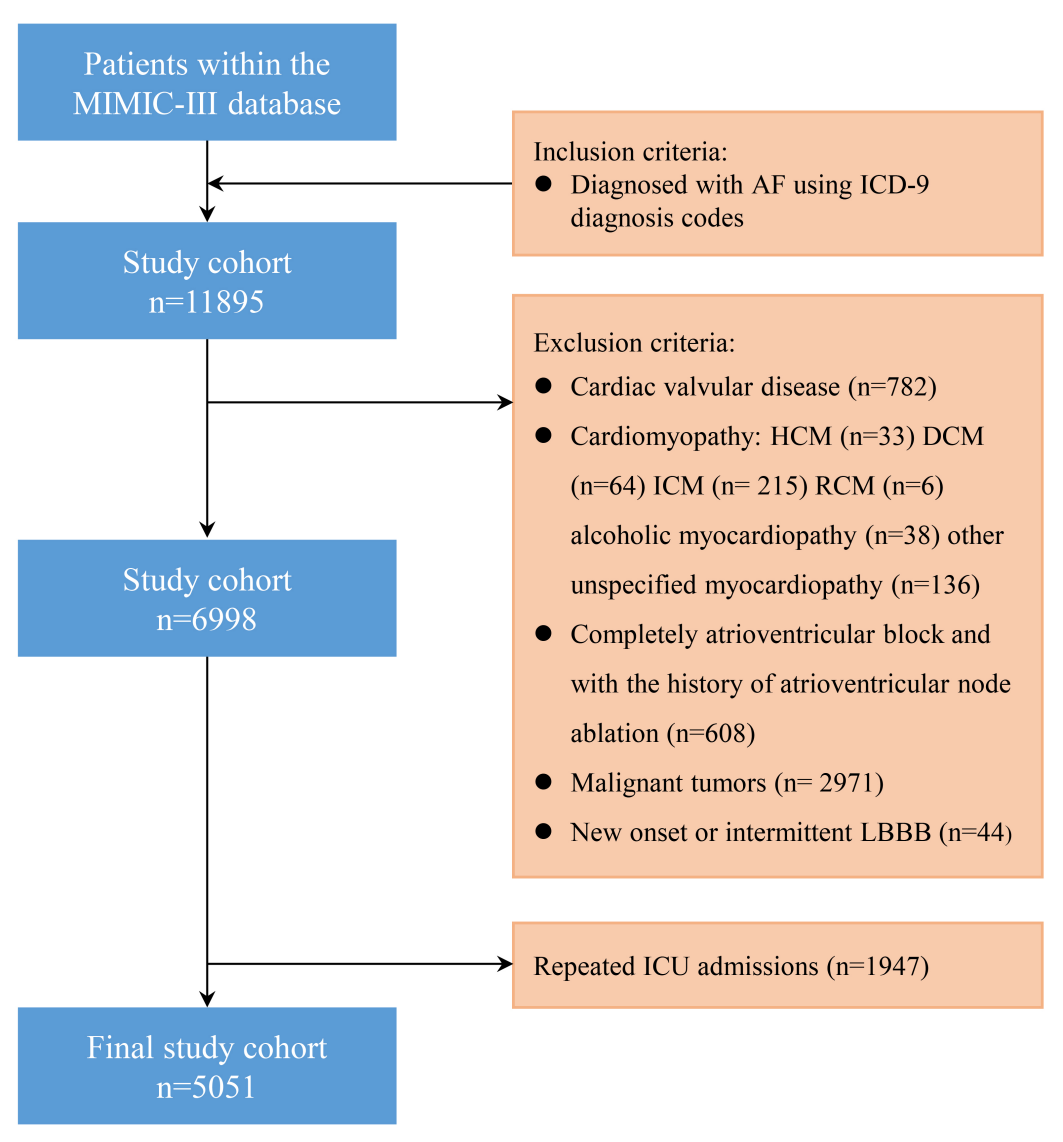
**Flow diagram of patient inclusion**. MIMIC-III, Medical Information Mart for Intensive Care-III; ICU, intensive care unit; ICD-9, International Classification of Diseases, Ninth Revision; HCM, hypertrophic cardiomyopathy; DCM, dilated cardiomyopathy; ICM, ischemic cardiomyopathy; RCM, restrictive cardiomyopathy; LBBB, left bundle branch block; AF, atrial fibrillation.

**Fig. 2.**
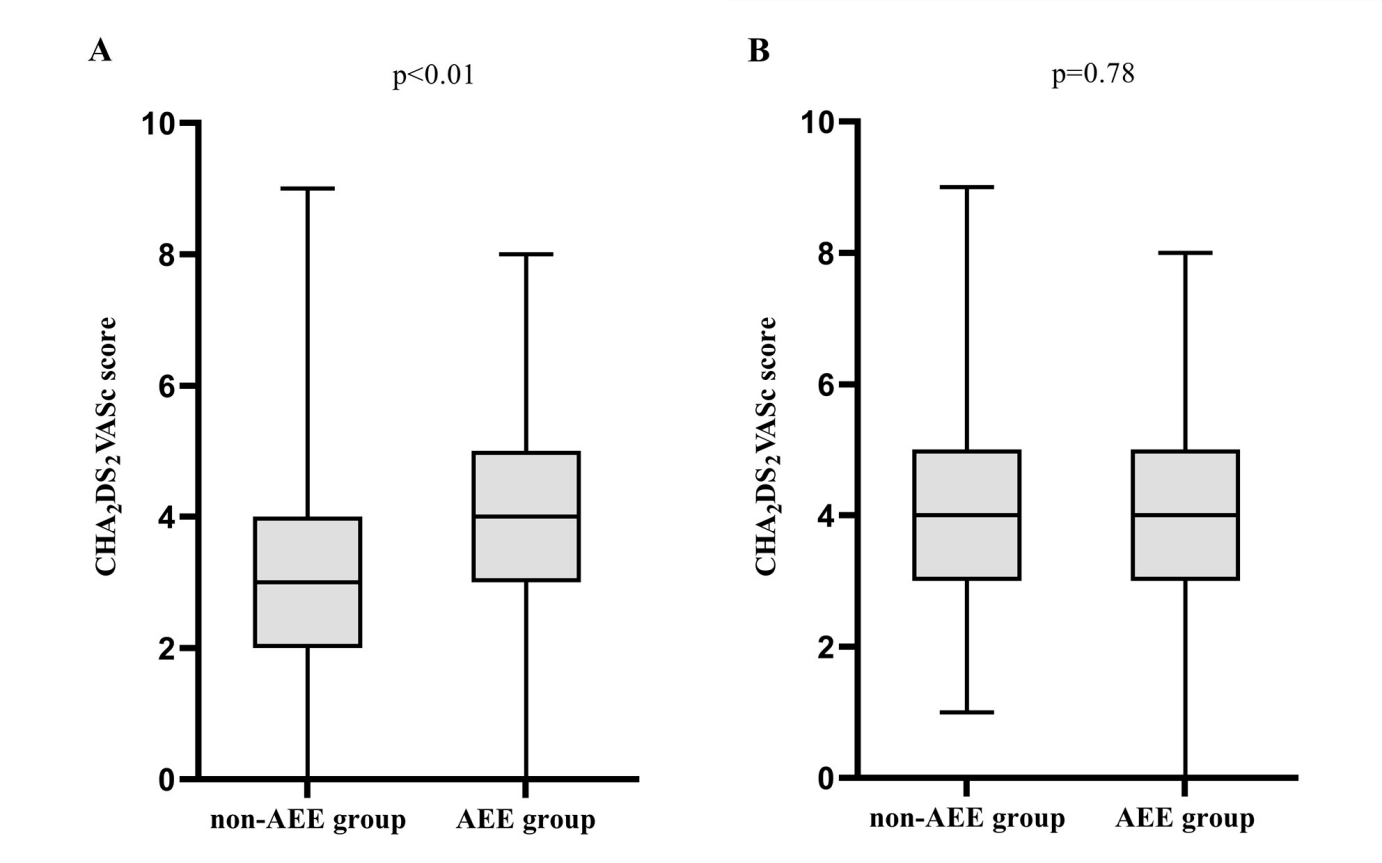
**The comparison of the CHA_2_DS_2_VASc score of patients with and without AEE in the non-LBBB group (A) and LBBB group (B)**. Mann–Whitney U test was used to compare the CHA_2_DS_2_VASc score. AEE, arterial embolism events; LBBB, left bundle branch block. The CHA_2_DS_2_VASc score, one point for chronic heart failure, hypertension, age 65-75 years, diabetes, vascular disease, female respectively; two points for age ≥75 years and stroke.

In total, 5051 patients were enrolled (Fig. 1). Among them, 65 patients had chronic LBBB (LBBB group), which accounted for 1.3% of the included population. The baseline characteristics of the two groups are summarized in Table 1. Patients with LBBB tended to be older, have a lower BMI and were mostly smokers. There were no significant differences regarding comorbidities such as hypertension, diabetes, CAD, stroke/TIA, chronic kidney disease. The LBBB group had more AF-related complications than non-LBBB group of both AEE (13.8% vs 3.8%, *p* = 0.02) and HF (64.6% vs 46.9%, *p* = 0.02). Though with higher CHA_2_DS_2_VASc score, 2 low risk patients (the score ≤2) had AEE in LBBB group, whereas no low risk patients had AEE in the non-LBBB group. The incidence of AEE was significant and positively correlated with CHA_2_DS_2_VASc score in the non-LBBB group (*p* < 0.01), but the tendency seemed to have disappeared in the LBBB group (*p* = 0.78) (Fig. 2). The in-hospital mortality was 12.7% and 12.3% in the non-LBBB and LBBB group respectively, without significant difference (*p* = 0.93).

**Table 1.** **Characteristics of the study patients before PSM**.

Characteristics		Non-LBBB group (n = 4986)	LBBB group (n = 65)	*p* value
Demographics				
	Age, years	71 (63, 78)	75 (65, 80)	<0.01
	Sex, male, n (%)	1832 (36.7%)	30 (46.1%)	0.12
	Nicotine dependence, n (%)	978 (19.6%)	18 (27.7%)	0.03
	BMI^a^	29.1 (25.2, 34.5)	27.5 (23.0, 30.7)	0.03
	Alcohol abuse, n (%)	211 (4.4%)	3 (4.6%)	1.00
	AF type, non-persistent AF*, n (%)	1839 (36.9%)	26 (40.0%)	0.72
	Heart rate in persistent AF, (bpm)	87 (75, 104)	95 (80, 120)	0.06
	CHA_2_DS_2_VASc score	3 (2, 4)	4 (3, 5)	<0.01
Comorbidities, n (%)				
	Hypertension	3644 (73.1%)	49 (75.4%)	0.78
	Diabetes	1869 (37.5%)	29 (46.0%)	0.25
	CAD	1191 (23.9%)	22 (33.8%)	0.06
	Stroke/TIA	410 (8.2%)	4 (6.2%)	0.55
	Chronic kidney disease	1215 (24.4%)	20 (30.8%)	0.23
AF related complications, n (%)				
	AEE^#^	338 (6.8%)	9 (13.8%)	0.04
	AEE in the patients of CHA_2_DS_2_VASc score ≤2	81 (4.84%)	2 (14.3%)	0.15
	Heart failure	1738 (34.8%%)	42 (64.6%)	<0.01
Laboratory-based data (blood)				
	Glucose, mg/dL	143.0 (111.0, 200.0)	141.0 (92.5, 177.0)	0.42
	hemoglobin, g/dL	10.7 (9.1, 12.4)	10.9 (10.0, 12.8)	0.35
	Troponin T, ng/mL	0.10 (0.03, 0.32)	0.11 (0.05, 0.62)	0.26
	Creatinine^b^, mg/dL	1.4 (0.9, 2.4)	1.6 (1.0, 2.6)	0.02
	Urea nitrogen, mg/dL	30.0 (19.0, 49.0)	33.0 (17.5, 60.0)	0.11
	LVEF^c^, %	55 (55, 55)	34 (26, 52)	<0.01
	In-hospital death, n (%)	633 (12.7%)	8 (12.3%)	0.93

PSM, propensity score matching; LBBB, left bundle branch block; BMI, body mass index; AF, atrial fibrillation; bpm, beats per minute; CAD, coronary artery disease; TIA, transient ischemic attack; AEE, arterial embolism events; LVEF, left ventricular ejection fraction; *: including paroxysmal AF and patients who accepted the rhythm control therapy; ^#^: including intracardiac thrombosis, cerebral infarction and peripheral embolism events, and, the number was 15, 289, 34 and 1, 8, 0 in the non-LBBB group and LBBB group respectively; ^a^: the default number was 1996 and 25 in the non-LBBB group and LBBB group respectively; ^b^: the default number was 84 and 0 in the non-LBBB group and LBBB group respectively; ^c^: the default number was 3527 and 48 in the non-LBBB group and LBBB group respectively.

### 3.2 Prognostic Significance of LBBB after PSM

In total, 65 pairs of the CHA_2_DS_2_VASc score and AF type matched patients were generated after 1:2 PSM (LBBB vs non-LBBB group). The baseline characteristics after PSM are illustrated in Table 2. After PSM, the LBBB group still had more AEE (13.8% vs 3.8%, *p* = 0.02), even in patients with the CHA_2_DS_2_VASc score ≤2 (14.3% vs 0, *p = *0.01). The multivariate analysis showed that LBBB was significantly and positively associated with AEE (OR 6.38, 95% CI [1.10, 36.93], *p* = 0.04) (Table 3). There was no significant difference in in-hospital mortality between the two groups, however, cardiac death happened more frequently in the LBBB group (7.7% vs 1.5%, *p* = 0.04). The results of logistic regression analysis for the in-hospital cardiac death were shown in Table 4. Age, LVEF, blood urea nitrogen and LBBB were the relevant factors obtained from the univariate analysis. No independent correlation factor was found through the multivariate analysis, however, when the LVEF was excluded from the model, the OR of LBBB increased markedly (from OR 1.73, 95% CI [0.05, 55.71], *p* = 0.76 to OR 7.53, 95% CI [0.82, 69.08], *p* = 0.07).

**Table 2.** **Characteristics of the study patients after PSM**.

Characteristics			non-LBBB group (n = 130)	LBBB group (n = 65)	*p* value
Demographics					
	Age, years		71 (65, 78)	75 (65, 80)	0.07
	Sex, male, n (%)		61 (46.9%)	30 (46.1%)	0.90
	Nicotine dependence, n (%)		31 (23.8%)	18 (27.7%)	0.56
	BMI^a^		29.1 (26.0, 36.4)	27.5 (23.0, 30.7)	0.03
	Alcohol abuse, n (%)		5 (3.8%)	3 (4.6%)	1.00
	AF type, non-persistent AF*, n (%)		48 (36.9%)	26 (40.0%)	0.68
	Heart rate in persistent AF, (bpm)		93 (81, 109)	90 (77, 106)	0.33
	CHA_2_DS_2_VASc score		4 (3, 5)	4 (3, 5)	0.63
Medications on admission, n (%)					
	Oral anticoagulants		40 (30.8%)	26 (40.0%)	0.20
	Beta-blocker		82 (63.6%)	36 (55.4%)	0.27
	Antiarrhythmic agents		23 (17.8%)	21 (32.8%)	0.07
		Amiodarone	4 (17.4%)	11 (52.4%)	0.01
		Dronedarone	0 (0%)	1 (4.8%)	0.48
		Diltiazem	13 (56.5%)	9 (42.8%)	0.36
		Verapamil	6 (26.0%)	0 (0%)	0.02
	ACEI/ARB		59 (5.7%)	39 (60.9%)	0.05
Comorbidities, n (%)					
	Hypertension		106 (81.5%)	49 (75.4%)	0.32
	Diabetes		57 (43.8%)	29 (46.0%)	0.92
	CAD		38 (29.2%)	22 (33.8%)	0.51
	Stroke/TIA		19 (14.6%)	4 (6.2%)	0.21
	Chronic kidney disease		45 (34.6%)	20 (30.8%)	0.59
AF related complications, n (%)					
	AEE^#^		5 (3.8%)	9 (13.8%)	0.02
	AEE in the patients of CHA_2_DS_2_VASc score ≤2		0 (0%)	2 (14.3%)	0.11
	Heart failure		61 (46.9%)	42 (64.6%)	0.02
Laboratory-based data(blood)					
	Glucose, mg/dL		161.0 (116.0, 199.0)	141.0 (92.5, 177.0)	0.48
	hemoglobin^b^, g/dL		9.4 (8.1, 11.9)	10.9 (10.0, 12.8)	<0.01
	Troponin T, ng/mL		0.07 (0.03, 0.46)	0.11 (0.05, 0.62)	0.19
	Creatinine, mg/dL		1.5 (1.0, 2.3)	1.6 (1.0, 2.6)	0.12
	Urea nitrogen, mg/dL		28.0 (20.0, 45.7)	33.0 (17.5, 60.0)	0.21
	LVEF^c^, %		55 (55, 55)	34 (26, 52)	<0.01
	In-hospital death, n (%)		15 (11.5%)	8 (12.3%)	0.87
	In-hospital cardiac death, n (%)		2 (1.5%)	5 (7.7%)	0.04

PSM, propensity score matching; LBBB, left bundle branch block; BMI, body mass index; AF, atrial fibrillation; bpm, beats per minute; CAD, coronary artery disease; TIA, transient ischemic attack; AEE, arterial embolism events; LVEF, left ventricular ejection fraction; ACEI/ARB, angiotensin converting enzyme inhibitors/angiotensin receptor blocker; *: including paroxysmal AF and patients who accepted the rhythm control therapy; ^#^: all were cerebral infarction events except 1 intracardiac thrombosis in the non-LBBB group; ^a^: the default number was 53 and 25 in the non-LBBB group and LBBB group respectively; ^b^: the default number was 10 and 0 in the non-LBBB group and LBBB group respectively; ^c^: the default number was 79 and 48 in the non-LBBB group and LBBB group respectively.

**Table 3.** **The multivariate logistic regression analysis of the AEE after PSM**.

Parameter	OR (95% CI)	*p* value
Age	0.99 (0.92, 1.07)	0.84
BMI	0.98 (0.89, 1.08)	0.64
Antiarrhythmic agents	0.62 (0.10, 3.87)	0.61
ACEI/ARB	1.63 (0.34, 7.86)	0.54
Heart failure	2.69 (0.47, 15.43)	0.26
Hemoglobin	0.98 (0.67, 1.43)	0.92
LBBB	6.38 (1.10, 36.93)	0.04

AEE, arterial embolism events; PSM, propensity score matching; BMI, body mass index; ACEI/ARB, angiotensin converting enzyme inhibitors/angiotensin receptor blocker; LBBB, left bundle branch block; CI, confidence intervals; OR, odds ratios.

**Table 4.** **The logistic regression analysis of the in-hospital cardiac mortality after PSM**.

Parameter	Univariate analysis		Multivariate analysis	
	OR (95% CI)	*p* value	OR (95% CI)	*p* value
Age (year)	1.21 (1.02, 1.42)	0.02	1.24 (0.90, 1.71)	0.18
Hypertension	0.33 (0.07, 1.52)	0.15	/	/
LVEF (%)	0.92 (0.84, 0.99)	0.04	0.92 (0.83, 1.02)	0.14
BUN	1.03 (1.00, 1.06)	0.04	1.04 (0.98, 1.10)	0.16
Heart failure	5.63 (0.66, 47.6)	0.11	/	**/**
Coronary artery disease	1.72 (0.37, 7.95)	0.48	/	**/**
LBBB	5.33 (1.01, 28.28)	0.04	1.73 (0.05, 55.71)	0.76

PSM, propensity score matching; LVEF, left ventricular ejection fraction; LBBB, left bundle branch block; BUN, blood urea nitrogen; CI, confidence intervals; OR, odds ratios.

**Fig. 3.**
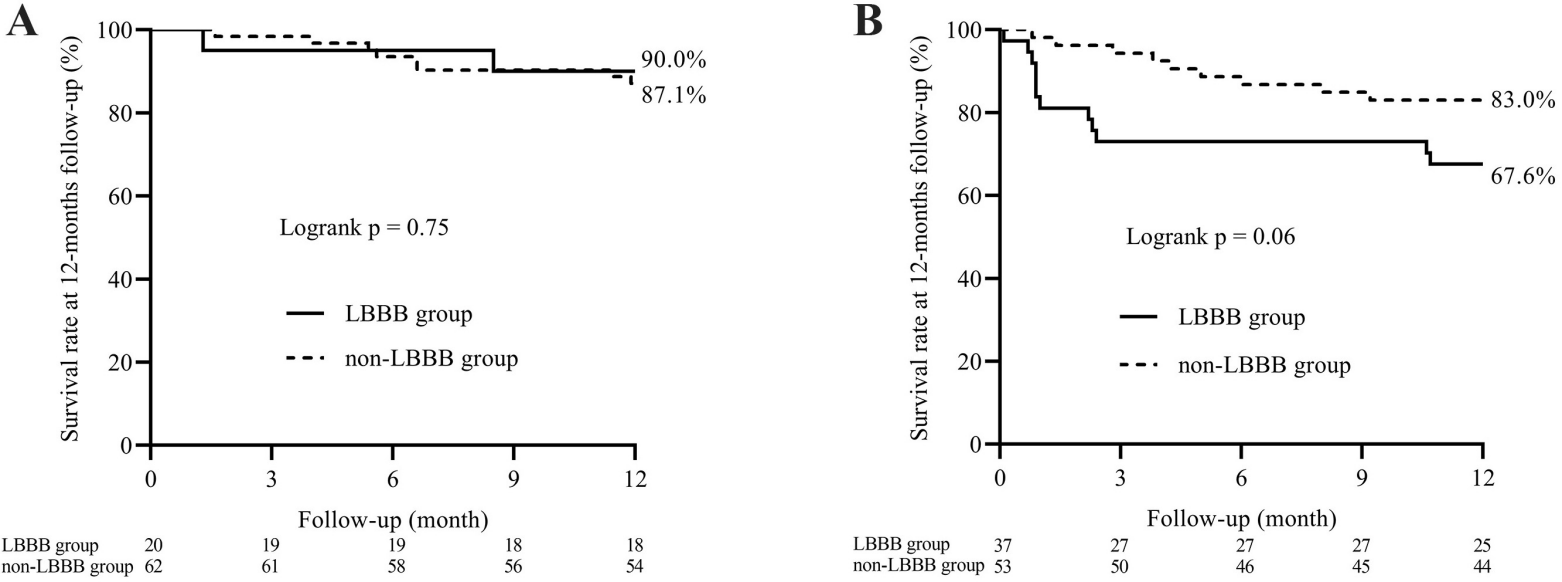
**Kaplan–Meier survival analysis plot for 1-year survival in the patients without (A) and with HF (B)**. The log-rank statistics were used for group comparison. LBBB, left bundle branch block; HF, heart failure.

The survival analysis showed that the LBBB group had a lower 1-year survival in the subgroup of HF (log-rank test: *p* = 0.06), and the difference disappeared in patients without HF (Fig. 3). A Cox regression model was performed to determine the risk factor of 1-year all-cause mortality. The result are summarized in Table 5: chronic kidney disease, HF and LBBB seemed to predict mortality together. Similarly, when the HF was excluded from the model, the HR of LBBB was raised (from HR 1.78, 95% CI [0.87, 3.65], *p* = 0.11 to HR 1.91, 95% CI [0.94, 3.89], *p* = 0.07).

**Table 5.** **The Cox regression analysis of 1-year survival after PSM**.

Parameter	Univariate analysis		Multivariate analysis	
	HR (95% CI)	*p* value	HR (95% CI)	*p* value
BMI	0.95 (0.89, 1.02)	0.14	/	/
AF type (Persistent AF)	1.37 (0.67, 2.80)	0.39	/	/
Heart rate (bpm)	0.99 (0.98, 1.01)	0.29	/	/
Usage of amiodarone	2.06 (0.46, 9.19)	0.34	/	/
Usage of verapamil	0.04 (0.0, 820.0)	0.53	/	/
Chronic kidney disease	2.14 (1.05, 4.31)	0.04	1.90 (0.91, 3.96)	0.09
BUN (mg/dL)	1.01 (1.00, 1.03)	0.09	/	/
Serum creatinine (mg/dL)	1.19 (1.06, 1.33)	<0.01	/	/
LVEF (%)	0.95 (0.91, 0.99)	0.04	/	*/*
Heart failure	2.12 (1.00, 4.51)	0.04	1.63 (0.74, 3.61)	0.22
LBBB	1.86 (0.91, 3.77)	0.09	1.78 (0.87, 3.65)	0.11

PSM, propensity score matching; BMI, body mass index; AF, atrial fibrillation; bpm, beats per minute; LVEF, left ventricular ejection fraction; LBBB, left bundle branch block; BUN, blood urea nitrogen; HR, hazard ratios; CI, confidence intervals.

## 4. Discussion

### 4.1 Main Findings

Our study investigated the association between chronic LBBB and the risk of AF-related endpoint events including AEE, HF and mortality among critically ill patients. The main findings were: (1) LBBB was a risk factor of AEE being independent from the CHA_2_DS_2_VASc score and HF; (2) LBBB was related to in-hospital cardiac death and 1-year all-cause mortality maybe through promoting the occurrence and progression of HF.

### 4.2 LBBB and AF

LBBB and AF have obvious clinical symbiosis tendencies. Based on the AF cohort from the AFBAR study (AF in the BARbanza area, a prospective study, n = 777 patients), the proportion of LBBB was as high as 8.8% in patients with AF in the absence of HF or left ventricular dysfunction [10]. Another result from the Nationwide Inpatient Sample (NIS) database between the years 2009 and 2015 was 1.7% [8]. While the data from the Chinese AF Registry (CAFR) (a prospective, multicenter, ongoing registry study) released recently was 0.36% [10], our study shows it was 1.3%. Similarly, patients with LBBB are more likely to develop AF [10, 11]. The mechanism of this symbiosis is not clearly demonstrated. AF and LBBB may both develop as a consequence of degenerative changes in the heart or various types of cardiomyopathy which affects both the atria and the ventricle [8, 14, 15]. On the other hand, the high frequency bombarding of AF impulses can cause electrophysiological remodeling of the AV node, slowing down its conduction, the similar impact on bundle branches may exist as well [8]. AF or fast heart rate-dependent LBBB is a common emergent situation causing hemodynamic disturbance or acute decompensated HF [16, 17], and vice versa. LBBB was reported to be related to functional mitral regurgitation, which, of course can lead to left atrial remodeling and AF [18, 19].

### 4.3 The Additive Effects on Prognosis

AF alone and isolated LBBB can both lead to cardiomyopathy and being independent predictors of malignant clinical outcomes [1, 20]. However, there is still controversy over their additive effects. Caught in the chicken and egg problem, the diagnosis of AF/LBBB-induced cardiomyopathy is always dependent on post-hoc analysis. But, some previous studies, and this study, did find that AF and LBBB co-exist in HF patients [14, 21]. Li W *et al*. [17] firstly reported a case that LBBB promoted the AF-induced cardiomyopathy, and AF catheter ablation reversed the cardiomyopathy which has been called “the AF- and LBBB-induced cardiomyopathy”. Alternatively, AF may be one of the triggers/predictors for LBBB-induced cardiomyopathy. The HF patients with LBBB seem to benefit from heart rate reduction more easily [22, 23]. The AF with rapid ventricular rate would aggravate the myocardial remodeling related to LBBB just like the burden in premature ventricular complexes (PVC)-induced cardiomyopathy. Another interesting case report shows that the cardiomyopathy was completely reversed due to LBBB correction by AF ablation and pharmacologic heart rate control in a patient with AF/high rate-dependent LBBB [24]. Cardiac resynchronization therapy (CRT) which works by pacing combined with atrioventricular junction ablation, is a highly effective treatment for HF patients with underlying AF [25]. However, the patients with both AF and LBBB seem to benefit more than patients with only one of them [26, 27]. So far, only a few studies have shown that LBBB is an independent risk factor of all-cause mortality in AF patients with HF but not in ones without HF [10, 11, 28], which is consistent with our results. Nevertheless, our study shows that LBBB, not LVEF, is independently correlated with in-hospital cardiac mortality in these critically ill patients with AF. A largest study to date [10] which aimed to explore the impact of LBBB on the outcome of AF patients admitted for catheter ablation (19% with HF) showed that LBBB was associated with a higher risk of a composite endpoint of stroke, all-cause mortality, and cardiovascular hospitalization. However, they did not rule out that it was attributable to higher AF recurrence rate in the LBBB group. Besides, our study is the first to find out that AF patients with LBBB have a higher risk of the AEE, which are not related to the CHA_2_DS_2_VASc score or HF. The mechanism is unknown. Maybe the hemodynamic abnormality caused by LBBB [18, 19] promotes intracardiac thrombosis in AF patients. Whether these patients need routine anticoagulation therapy or LBBB rectification (left bundle branch pacing) which could then lower the risk of AEE should be studied further.

### 4.4 Limitations

Some limitations of our study should be discussed. Firstly, this is a retrospective single-center study with the clinical data of patients admitted to ICU who often have more severe comorbidities or advanced disease, which might limit the generalization of our findings. Secondly, the sample size of the LBBB group was rather small (65 patients), which may decrease statistical efficiency and bring some bias with PSM. For the same reason, we included just the CHA_2_DS_2_VASc score (the key factor of both stroke and all-cause mortality) and AF type (the overlap burden of LBBB and AF may be the key) into PSM. So, several covariates were unbalanced between the groups (e.g., BMI, HF). However, the tendency was the same as it was before PSM (Tables 1,2), the selection bias brought by PSM could be minimal. We further performed multiple regression analyses. Thirdly, the impact of the different provisional diagnoses and therapy on the outcome was not taken into consideration. Lastly, the LBBB might be diagnosed by non-cardiologists which could be heterogenous including true LBBB and interventricular/intraventricular conduction block with an LBBB-like pattern.

## 5. Conclusions

Through a retrospective analysis of the non-valvular AF cohort from the MIMIC-III database, we demonstrated that LBBB is an independent risk factor for adverse outcomes of AF, including AEE and the onset and progression of HF.

## Availability of Data and Materials

The datasets used and/or analyzed during the current study are available from the corresponding author on reasonable request.
